# Supplementary material for: Development of a Modified QuEChERS Method Based on Magnetic Multi-Walled Carbon Nanotubes as a Clean-Up Adsorbent for the Analysis of Heterocyclic Aromatic Amines in Braised Sauce Beef
Source: Foods. 2022 Dec 27;12(1):138. doi: 10.3390/foods12010138 (PMC9818259; doi:10.3390/foods12010138)
Supplement: Supplementary file 1 [file foods-12-00138-s001.zip › Supplementary Materials.pdf]

Development of a Modified QuEChERS method based on Magnetic Multiwalled Carbon Nanotubes as a Clean-up Adsorbent for the Analysis of Heterocyclic Aromatic Amines in Braised Sauce Beef

Min Li [1], Pengxiang Wang [1], Xu Zhang [1], Hongyu Wang [1], Ke Li [1], Yanhong Bai [1,2,*]

[1] College of Food and Bioengineering, Zhengzhou University of Light Industry, Zhengzhou 450001, China

[2] Henan Key Laboratory of Cold Chain Food Quality and Safety Control, Zhengzhou 450001, China

[*] Correspondence: baiyanhong212@163.com

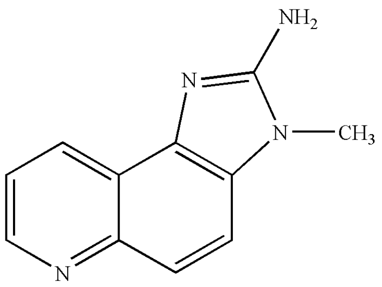

IQ

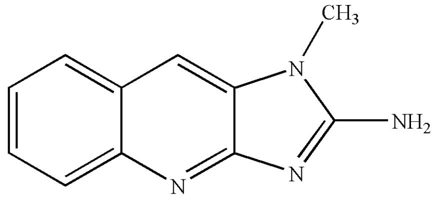

IQ[4,5-b]

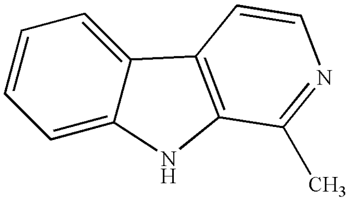

Harman

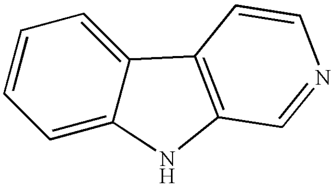

Norharman

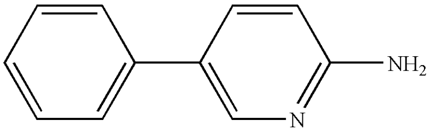

Phe-P-1

Figure S1. Chemical structures of the studied HAAs.

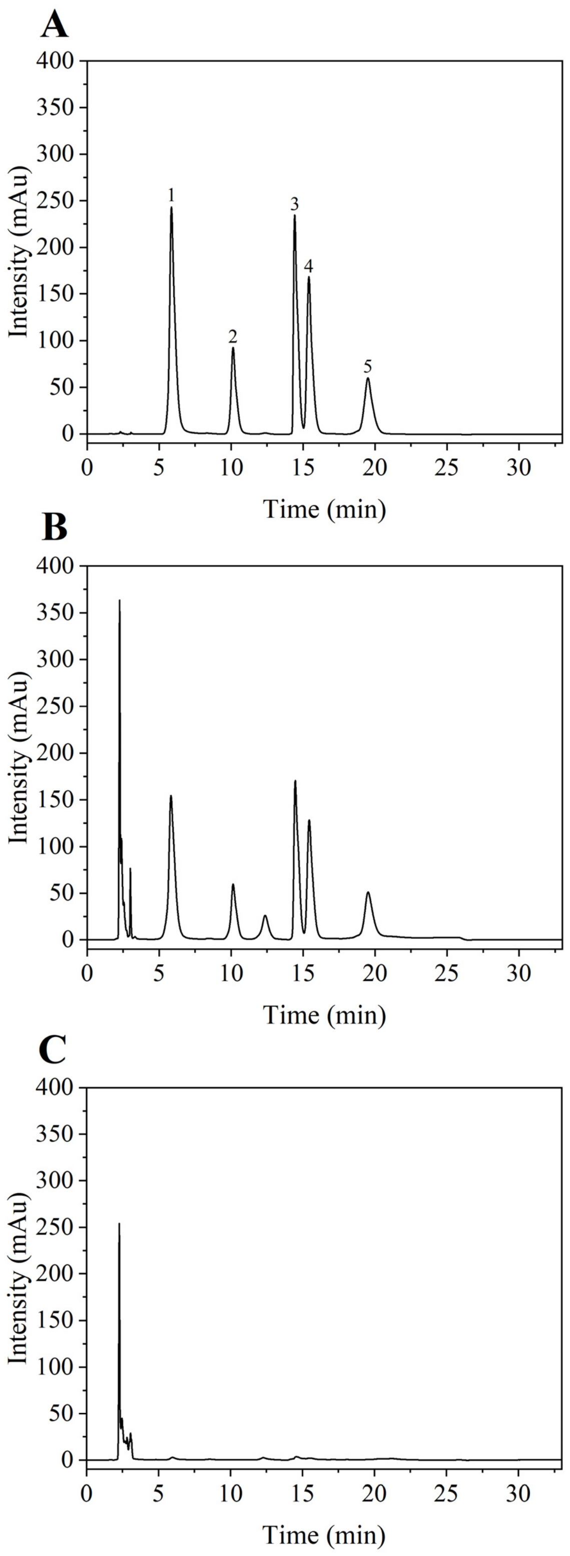

Figure S2. Chromatograms of the mixed standard solution (**A**), the spiked braised sauce beef extract (**B**) and the blank braised sauce beef extract (**C**) prepared with the proposed QuEChERS method. Peak assignments: (1) IQ, (2) IQ[4,5-b], (3) Harman, (4) Norharman, (5) Phe-P-1. Spiked concentrations, 1.0 μg/g for IQ, Harman and Norharman, 0.5 μg/g for IQ[4,5-b] and Phe-P-1.

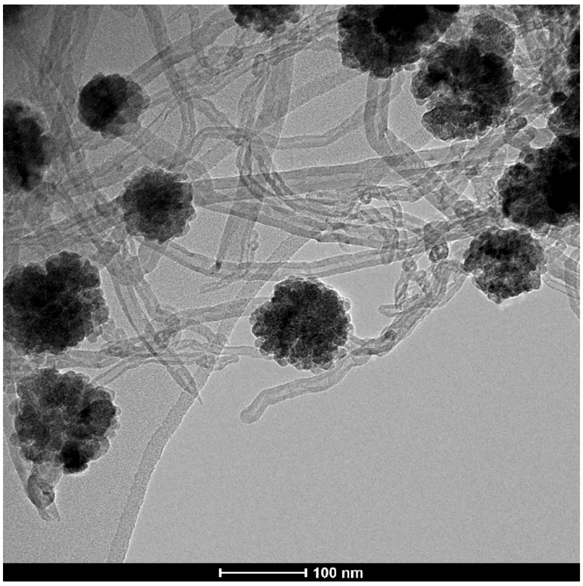

Figure S3. TEM image of Fe_3O_4-MWCNTs.

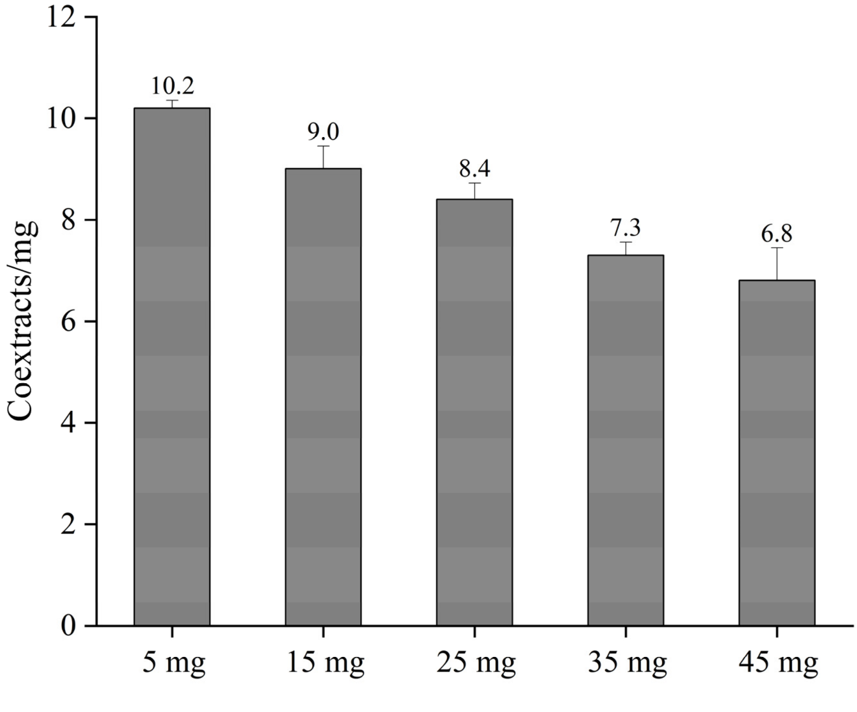

Figure S4. Amount of co-extracted matrix obtained through gravimetric analysis after purification with different amounts of Fe_3O_4-MWCNTs in the QuEChERS method (n = 3).

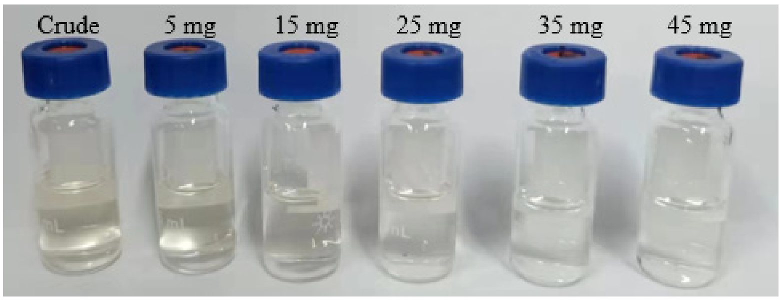

Figure S5. Photographs of braised sauce beef extract after purification with different amounts of Fe_3O_4-MWCNTs.

Table S1. HAAs measured in analyzed braised sauce beef samples.

Samples	IQ	IQ[4,5-b]	Harman	Norharman	Phe-P-1
Beef 1	n.q. [1]	-	n.q.	n.q.	-
Beef 5	n.q.	-	-	-	-
Beef 8	n.q.	-	n.q.	n.q.	-
Beef 13	n.q.	-	n.q.	n.q.	-
Beef 14	n.q.	-	n.q.	n.q.	-

[1] n.q.-not quantified (<LOQ), HAAs could be detected but not quantified.
